# Clinical and therapeutic features and prognostic factors of metastatic colorectal cancer over age 80: a retrospective study

**DOI:** 10.1186/s12876-021-01791-9

**Published:** 2021-05-01

**Authors:** Hiroyuki Hisada, Yu Takahashi, Manabu Kubota, Haruhisa Shimura, Ei Itobayashi, Kenji Shimura, Akira Nakamura

**Affiliations:** 1 Department of Gastroenterology, Graduate School of Medicine, the University of Tokyo, 7-3-1 Hongo, Bunkyo-ku, Tokyo, 113-8655 Japan; 2 Department of Gastroenterology, Asahi General Hospital, Chiba, Japan

**Keywords:** Colorectal cancer, Elderly, Prognostic factor, Nutritional status, Chemotherapy

## Abstract

**Background:**

Colorectal cancer (CRC) is one of the most common cancers in the world. The number of elderly patients with CRC increases due to aging of the population. There are few studies that examined chemotherapy and prognostic factors in metastatic colorectal cancer (mCRC) patients aged ≥ 80 years. We assessed the efficacy of chemotherapy and prognostic factors among patients with mCRC aged ≥ 80 years.

**Methods:**

We retrospectively analyzed clinical and laboratory findings of 987 patients newly diagnosed with CRC at Asahi General Hospital (Chiba, Japan) between January 2012 and December 2016. The Kaplan–Meier method was used for the overall survival (OS) and the log-rank test was used to identify difference between patients. A multivariate Cox proportional hazard regression analysis was performed to determine the hazard ratios and 95% confidence intervals (CIs) of prognostic factors among super-elderly patients.

**Results:**

In total, 260 patients were diagnosed with mCRC (super-elderly group: n = 43, aged ≥ 80 years and younger group, n = 217, aged < 80 years). The performance status and nutritional status were worse in the super-elderly group than in the younger group. The OS of super-elderly patients who received chemotherapy was worse than that of younger patients (18.5 vs. 28.8 months; P = 0.052), although the difference was not significant. The OS of patients who received chemotherapy tended to be longer than that of those who did not; however, there were no significant differences in OS in the super-elderly group (18.5 vs. 8.4 months P = 0.33). Multivariate analysis revealed that carcinoembryonic antigen levels ≥ 5 ng/mL (hazard ratio: 2.27; 95% CI 1.09–4.74; P = 0.03) and prognostic nutritional index ≤ 35 (hazard ratio: 8.57; 95% CI 2.63–27.9; P = 0.0003) were independently associated with poor OS in the super-elderly group.

**Conclusions:**

Patients with mCRC aged ≥ 80 years had lower OS than younger patients even though they received chemotherapy. Carcinoembryonic antigen and prognostic nutritional index were independent prognostic factors in super-elderly patients with mCRC, but chemotherapy was not.

*Trial registration*: retrospectively registered.

**Supplementary Information:**

The online version contains supplementary material available at 10.1186/s12876-021-01791-9.

## Background

Colorectal cancer (CRC) is the third most common cancer worldwide, and about 82% of patients diagnosed with sporadic colon cancer are aged ≥ 60 years [1]. The median age of patients diagnosed with CRC in developed countries is 70 years. Among patients aged ≥ 80 years in the United States, approximately 19% are men and 29% women [2]. The life expectancy of a Japanese 80-year-old was 88.9 years in men and 91.8 years in women in 2017 [3]. The proportion of elderly patients who undergo surgery, receive chemotherapy, or both increases in clinical practice [4]. However, old age is considered a risk factor for complications because elderly patients usually have comorbidities and are frail [5]. Due to advancements in chemotherapy, the prognosis of young patients with unresectable metastatic CRC (mCRC) has improved, but that of elderly patients with mCRC has not improved [6]. Standard chemotherapy regiments for mCRC are based on doublet chemotherapy agents, such as fluoropyrimidine plus oxaliplatin or irinotecan combined with a molecular-targeted agent (bevacizumab, cetuximab, or panitumumab) for healthy patients. Meanwhile, the efficacy of combination chemotherapy based on oxaliplatin or irinotecan in super-elderly patients remains unelucidated. Therefore, chemotherapy is often administered to remove oxaliplatin or irinotecan from standard treatment in elderly patients according to a randomized phase 3 trial [7].

Several studies have attempted to evaluate chemotherapy in elderly patients. However, most of them have included elderly patients aged ≥ 65 years with mCRC, and others have included patients aged ≥ 70 years or patients of different ages [7–9]. Moreover, super-elderly patients (aged ≥ 80 years) have worse performance status (PS) and more comorbidities than younger patients. Clinical trials cautiously select participants (with the application of age restriction during study enrolment and with consideration of patients with good PS and comorbidities); therefore, elderly patients are significantly under-represented [10–12]. Only few studies showed the clinical characteristics of patients with mCRC aged ≥ 80 years and the survival benefits of surgical resection and chemotherapy in these patients [13–15].

Primary tumor resection, carcinoembryonic antigen (CEA), chemotherapy, microsatellite instability, *RAS*-*BRAF* mutation, and prognostic nutritional index (PNI) have been identified as the prognostic factors of mCRC [16–18]. However, few studies have analyzed prognostic factors specifically for the super-elderly patients.

Hence, the current study aimed to investigate the efficacy of chemotherapy and whether the prognostic factors described in previous reports apply to super-elderly patients with mCRC.

## Methods

This study was approved by the institutional review board of Asahi General Hospital (approval no. 2019031914). Due to the use of anonymous data, instead of obtaining informed consent, information about the study, and the option to drop out was provided to the participants. This study was performed in accordance with the standards of the Declaration of Helsinki and the current ethical guidelines. This was a cross-sectional retrospective study, and all medical records were retrospectively reviewed.

Patients whose CRC diagnosis was confirmed between January 2012 and December 2016 based on histological examination or colonoscopy and radiologic images, such as computed tomography (CT) and magnetic resonance imaging (MRI), were included. Patients with localized colorectal cancer and radical R0 resections were excluded. Staging was performed using the International Union for Cancer Control TNM classification 7th ed.

The diagnosis of CRC was confirmed on colonoscopy and pathological examinations and on imaging studies, such as CT and MRI. We analyzed the medical records until December 2019.

### Data collection

Data on the characteristics of the patients, including age, sex, tumor location, metastatic site, Eastern Cooperative Oncology Group PS score, histopathology reports, serum CEA level, body mass index (BMI), creatinine and albumin levels, PNI, chemotherapy, and OS, were extracted. Serum samples were collected and tested when the patients were diagnosed with mCRC. OS was defined as the period from the diagnosis of mCRC to the date of death or last follow-up. Adverse events were defined according to the National Cancer Institute-Common Toxicity Criteria version 4.0. Eastern Cooperative Oncology Group PS score was defined based on a previous study [19]. Poorly differentiated cancer was defined as tumor with 5%–50% glandular structure, and signet-ring cell carcinoma was defined as the presence of > 50% of tumor cells with prominent intracytoplasmic mucin [20]. The PNI was calculated as follows: 10 × albumin value (g/dL) + 0.005 × TLC of the peripheral blood, where TLC is the total lymphocyte count/mm^3^. BMI was classified as either < 18.5 or ≥ 18.5 kg/m^2^ and PNI as either as either < 35 and ≥ 35, based on previous reports [21, 22]. Patients with cecum, ascending colon, and transverse colon cancer were categorized under the right-side group, and those with descending, sigmoid colon, and rectal cancer were categorized under the left-side group [23].

### Statistical analysis

All statistical analyses were performed with EZR (Saitama Medical Center, Jichi Medical University, Saitama, Japan), which is a graphical user interface for R (The R Foundation for Statistical Computing, Vienna, Austria). Comparisons between groups were made using the Mann–Whitney U test for continuous and ordinal variables and the chi-square test for categorical variables. The Kaplan–Meier method was utilized to analyze the OS, and the log-rank test was used to identify differences between the patients. To assess the impact of some factors on the OS, univariate, and multivariate analyses of OS were performed using the Cox proportional hazard model. Data were summarized with hazard ratio along with 95% confidence interval (CI). The variables with P values < 0.10 in the univariate model were included in the multivariate model. Missing data were excluded from the analysis. All reported P values were two-tailed, and P values < 0.05 were considered statistically significant.

## Results

Between January 2012 and December 2016, 987 patients were newly diagnosed with CRC. Of them, 702 patients with localized colorectal cancer and 25 patients who received radical R0 surgery were excluded. Finally, 260 patients with mCRC were evaluated. We stratified the patients into two groups according to age at the time of diagnosis: the super-elderly group (n = 43, ≥ 80 years) and the younger group (n = 217, < 80 years).

A detailed flowchart of the inclusion and exclusion criteria is presented in Fig. S1 (in the Additional file 1).

### Characteristics of the participants

The clinical and pathological characteristics of the patients (n = 260) are presented in Table 1.

**Table 1:** Baseline Characteristics of patients diagnosed with mCRC, separated according to those aged < 80 and ≥ 80 years old

Variable	Younger group (n = 217)	Super elderly group (n = 43)	
	n or median (range)	n or median (range)	P
Age(years)	68 (27–79)	83 (80–93)	< 0.0001
Male sex (%)	135 (62%)	21 (49%)	0.11
PS score of 0–1	197 (91%)	28 (65%)	< 0.0001
Primary site			0.32
Colon	187 (86%)	40 (93%)	
Rectum	30 (14%)	3 (7%)	
Tumor located in the right-side	83 (38%)	11 (26%)	0.12
Site of metastases			
Liver only	80 (37%)	15 (35%)	0.86
Lung only	19 (9%)	7 (16%)	0.16
Liver and lung	35 (16%)	7 (16%)	1
Other	88 (41%)	14 (33%)	0.39
Albumin level (g/dL)	3.8	3.7	0.21
BMI	22.9	21.4	0.03
PNI	46.2	42.6	0.04
Creatinine level (mg/dL)	0.77	0.84	0.1
CEA level (ng/mL)	20.1	11.4	0.04
RAS mutation type	78 (46%)	7 (54%)	0.58
Poorly differentiated cancer or signet-ring cell carcinoma	14 (6%)	9 (21%)	< 0.0001

The proportion of patients with good PS was lower in the super-elderly group than in the younger group (65% vs. 91%; P < 0.0001). The BMI (21.4 vs. 22.9; P = 0.03) and PNI (42.6 vs. 46.2; P = 0.04) of the super-elderly group were lower than those of the younger group. The creatinine and albumin levels were not significantly different between the two groups. The proportion of patients with poorly differentiated cancer or signet-ring cell carcinoma was higher in the super-elderly group than in the younger group (21% vs. 6%; P < 0.0001).

### Treatment

No significant differences were found between the two groups in terms of the resection rate of primary and metastatic tumors. The number of patients who received chemotherapy in the super-elderly group was significantly lower than that in the younger group (28% vs. 84%; P < 0.0001).

These data are summarized in Table 2.

**Table 2:** Treatments administered to patients with mCRC, separated according to those < 80 and ≥ 80 years old

Variable	Younger group (n = 217)	Super-elderly group (n = 43)	
	n (range)	n (range)	P value
Resection of primary tumor	152 (70%)	32 (74%)	0.7
Chemotherapy	182 (84%)	12 (28%)	< 0.0001
No treatment	34 (16%)	31 (72%)	< 0.0001

### Chemotherapy

Only 12/43 patients (28%) in the super-elderly group received chemotherapy, and the prescribed chemotherapeutic agents were capecitabine, 5-FU/LV, oxaliplatin, bevacizumab, and panitumumab. Of them, 10 (83%) received combination chemotherapy, and the most common (n = 9, 75%) combination was a fluoropyrimidine plus oxaliplatin, with or without a molecular-targeted agent. Of the 12 patients who had first-line palliative chemotherapy, only 5 (42%) received chemotherapy for > 6 months, although most patients in the younger group (n = 152, 84%) received chemotherapy for > 6 months. In addition, only 5 (42%) patients and only 1 (8%) patient in the super-elderly group received second-line and third-line chemotherapy, respectively. Meanwhile, most (128) patients in the younger group (70%) and 78 (43%) patients received second-line and third-line chemotherapy, respectively. A summary of the chemotherapy regimens is presented in Table S1 (in the Additional files 2).

Patients who received chemotherapy had significantly better PS than those who did not receive it in the super-elderly group (100% vs. 34%; P = 0.006). Albumin levels and PNI of patients who received chemotherapy were higher than those who did not receive it in the super-elderly group (4.1 vs. 3.4 g/dl; P = 0.005 and 48.9 vs. 42.1; P = 0.009).

### Adverse events

Grade 3 or 4 adverse events in first-line chemotherapy were reported in 31.9% and 66.7% of patients in the younger and super-elderly groups, respectively (P = 0.02). The most frequent adverse events ≥ grade 3 in the younger group were neutropenia, with grade 3 or 4 neutropenia reported in 14.3% of patients; meanwhile, the most frequent adverse events ≥ grade 3 in the super-elderly group were fatigue, with grade 3 or 4 fatigue reported in 33.3% of patients. Meanwhile, super-elderly patients receiving mono-chemotherapy (n = 2) did not report any grade 3 or 4 adverse events. These data are summarized in Table 3.

**Table 3:** Adverse events, separated according to those < 80 and ≥ 80 years old

Variable	Younger group (n = 182)	Super-elderly group (n = 12)	
	n (range)	n (range)	P value
Any toxicity Grade ≥ 3	58 (31.9%)	8 (66.7%)	0.02
fatigue Grade ≥ 3	3 (1.6%)	4 (33.3%)	0.003
Anorexia Grade ≥ 3	6 (3.2%)	2 (16.7%)	0.08
Diarrhoea Grade ≥ 3	1 (0.5%)	0 (0%)	1
Neurosensory Grade ≥ 3	17 (9.3%)	0 (0%)	0.61
hand-foot syndrome Grade ≥ 3	2 (1.1%)	0 (0%)	1
Neutropenia Grade ≥ 3	26 (14.3%)	1 (8.3%)	1

### Prognostic factors of OS

The results of the univariate and multivariate analyses of OS in the super-elderly and younger groups are shown in Tables 4 and 5, respectively. The univariate analysis in the super-elderly group (Table 4) revealed that a PS score of 2–4, PNI ≤ 35, CEA level ≥ 5 ng/mL, and resection of primary tumors were significantly associated with OS. Based on the multivariate analysis, a CEA level ≥ 5 ng/mL (hazard ratio: 2.27; 95% CI 1.09–4.74; P = 0.03) and PNI ≤ 35 (hazard ratio: 8.57; 95% CI 2.63–27.9; P = 0.0003) were independently associated with poor OS.

**Table 4:** Analysis of prognostic factors in the super-elderly group

Variables	Univariate analysis		Multivariate analysis	
	Hazard ratio (95% CI)	P value	Hazard ratio (95% CI)	P value
Sex (male/female)	0.85 (0.43–1.66)	0.63		
PS score of 2–4	2.07 (0.99–4.29)	0.052	0.98 (0.35–2.75)	0.98
Tumor located on the right side	1.23 (0.59–2.57)	0.58		
Albumin level ≤ 3.5 g/dL	1.56 (0.80–3.07)	0.19		
BMI ≤ 18.5	1.74 (0.66–4.54)	0.26		
PNI ≤ 35	6.15 (2.00–18.9)	0.001	8.57 (2.63–27.9)	0.0003
Creatinine level ≥ 1.5 mg/dL	1.78 (0.61–5.20)	0.29		
CEA level ≥ 5 ng/mL	1.85 (0.91–3.77)	0.089	2.27 (1.09–4.74)	0.03
RAS mutation type	1.20 (0.36–4.02)	0.76		
Poorly differentiated cancer or signet-ring cell carcinoma	0.64 (0.28–1.51)	0.3		
Resection of primary tumor	0.41 (0.18–0.92)	0.026	0.45 (0.20–1.04)	0.06
Chemotherapy	0.68 (0.32–1.47)	0.33

**Table 5:** Analysis of prognostic factors in the younger group

Variables	Univariate analysis		Multivariate analysis	
	Hazard ratio (95% CI)	P value	Hazard ratio (95% CI)	P value
Sex (male/female)	1.09 (0.77–1.54)	0.63		
PS score of 2–4	3.49 (1.86–6.54)	< 0.0001	1.16 (0.57–2.35)	0.68
Tumor located on the right side	1.02 (0.72–1.44)	0.92		
Albumin level ≤ 3.5 g/dL	1.60 (1.13–2.26)	0.008	1.61 (1.13–2.28)	0.008
BMI ≤ 18.5	1.08 (0.62–1.88)	0.79		
PNI ≤ 35	1.38 (0.84–2.27)	0.21		
Creatinine level ≥ 1.5 mg/dL	0.62 (0.25–1.53)	0.30		
CEA level ≥ 5 ng/mL	1.35 (0.89–2.04)	0.16		
RAS mutation type	1.06 (0.72–1.54)	0.78		
Poorly differentiated cancer or signet-ring cell carcinoma	0.74 (0.38–1.46)	0.38		
Resection of primary tumor	0.45 (0.31–0.64)	< 0.0001	0.44 (0.31–0.64)	< 0.0001
Chemotherapy	0.23 (0.14–0.38)	< 0.0002	0.22 (0.13–0.37)	< 0.0001

In the multivariate analysis, resection of primary tumors (hazard ratio: 0.44; 95% CI 0.31–0.64; P < 0.0001) and chemotherapy (hazard ratio: 0.22; 95% CI 0.13–0.37; P < 0.0001) were identified as significant, independent, and good prognostic factors in the younger group (Table 5). By contrast, OS was significantly reduced when the albumin level was ≤ 3.5 g/dL (hazard ratio: 1.61; 95% CI 1.13–2.28; P = 0.008). These data are summarized in Tables 4 and 5.

### Survival

**Fig. 1:**
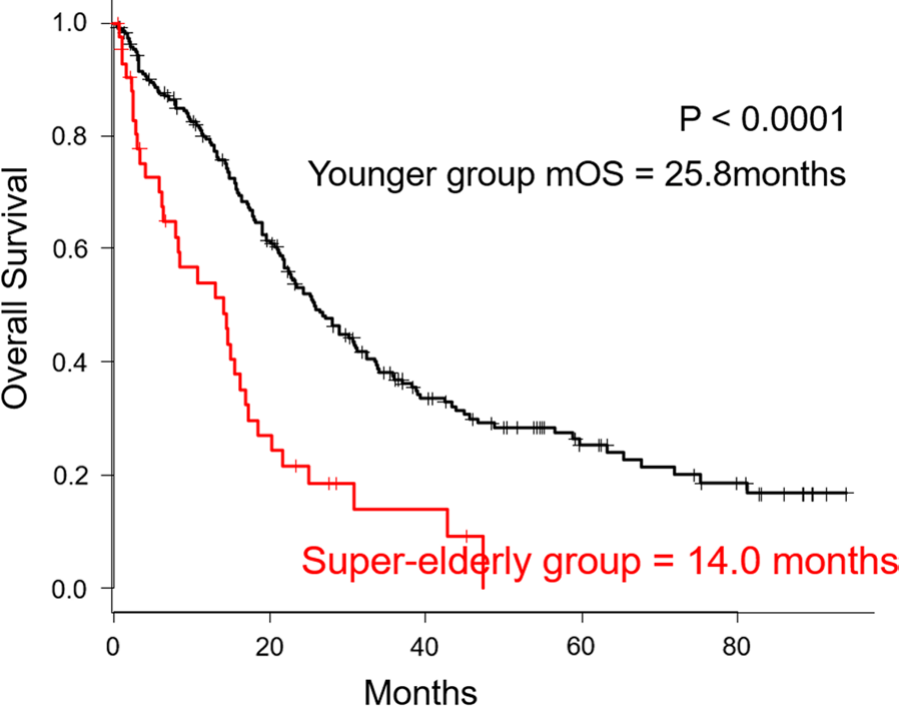
Kaplan–Meier curves comparing the super-elderly group with the younger group. The median OS was 14.0 months in the super-elderly group and 25.8 months in the younger group. (P < 0.0001). The 1- and 3-year OS in the in the younger group were 79% and 37%, respectively, and those in the super-elderly group were 54% and 14%, respectively

During the follow-up period, 35 (81%) patients in the super-elderly group and 140 (65%) in the younger group died. All 35 patients in the super-elderly group died of primary cancer. On the other hand, in the younger group, 132 patients died of primary cancer, 5 died of pneumonia, 1 died of acute arterial occlusion, 1 died of intestinal perforation, and 1 died of other cancers. There were significant differences in terms of the median OS between the two groups (14.0 vs. 25.8 months; P < 0.0001). The 1- and 3-year OS in the younger group were 79% and 37%, respectively, and those in the super-elderly group were 54% and 14%, respectively. These data are summarized in Fig. 1.

**Fig. 2:**
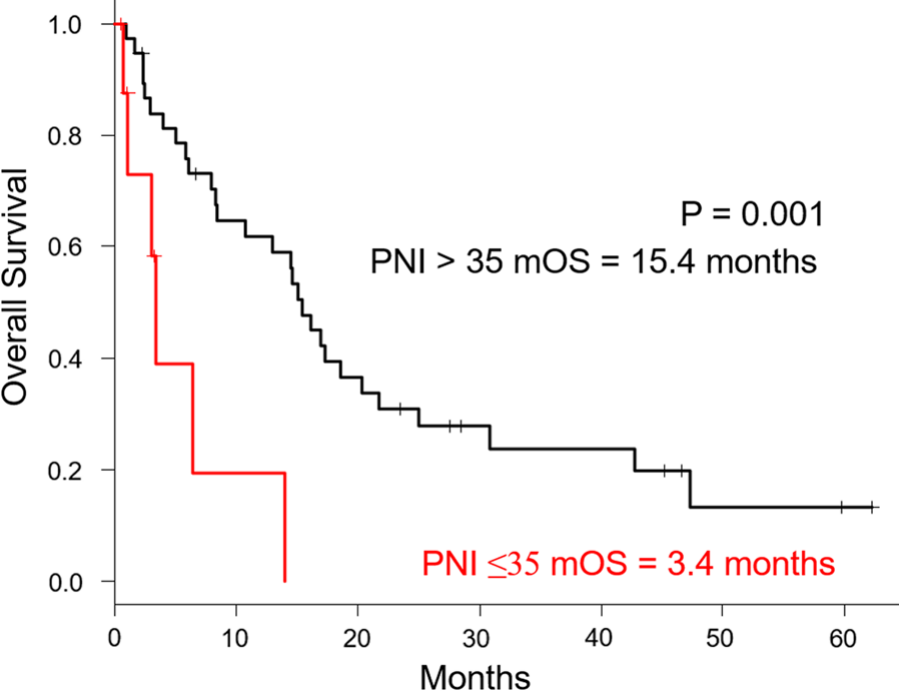
Kaplan–Meier curves comparing patients with PNI ≤ 35 and PNI > 35 in super-elderly patients. Patients with PNI ≤ 35 had was 3.4 months and Patients with PNI > 35 was 15.4 months in super-elderly patients. The median OS was significantly different between patients with PNI ≤ 35 and PNI > 35 (P = 0.001)

The median OS of patients in super-elderly patients was significantly different between patients with PNI ≤ 35 and PNI > 35 (3.4 months vs 15.4 months, P = 0.001). These data are summarized in Fig. 2.

**Fig. 3:**
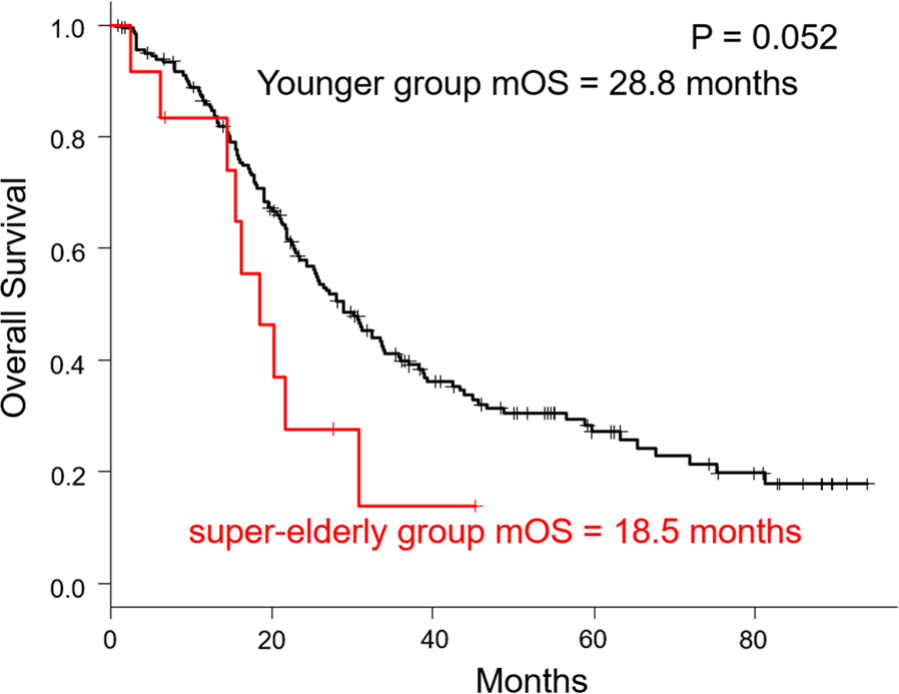
Kaplan–Meier curves comparing patients who received chemotherapy. The median OS of patients in the super-elderly group who received chemotherapy was 18.5 months, and that of patients in the younger group was 28.8 months, and the difference in median OS between the two groups approached significance (P = 0.052)

The median OS of patients in the super-elderly group who received chemotherapy was 18.5 months, and that of patients in the younger group was 28.8 months, and the difference in median OS between the two groups was close to significance (P = 0.052). These data are summarized in Fig. 3.

**Fig. 4:**
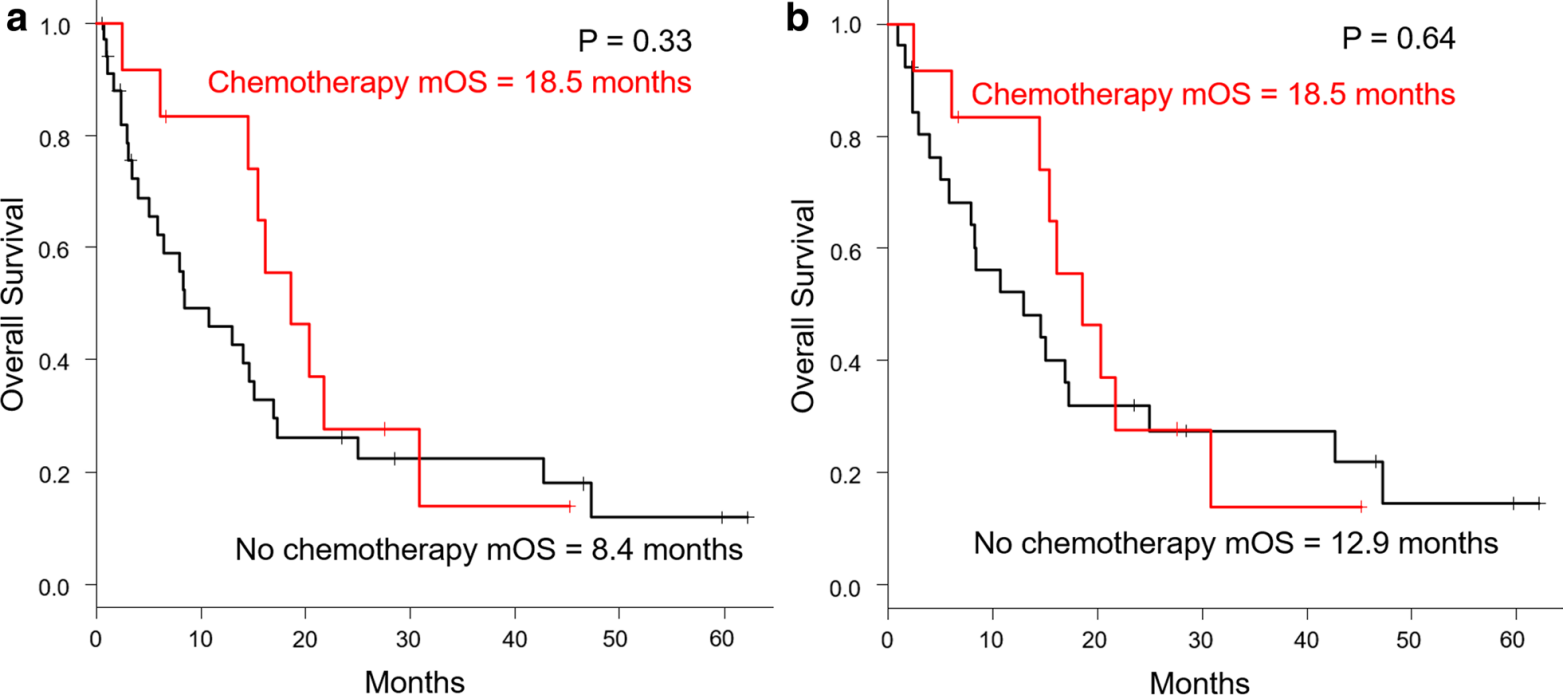
Kaplan–Meier curves of the super-elderly group patients who did and did not receive chemotherapy. There were no significant differences in the median OS between those who received chemotherapy and those did not receive it in the super-elderly group; however, OS of patients who received chemotherapy tended to be longer (18.5 vs. 8.4 months; P = 0.33). b: Kaplan–Meier curves of the super-elderly patients with PNI > 35. There were also no significant differences in the median OS in super -elderly patients with PNI > 35; however, OS of patients who received chemotherapy tended to be longer (18.5 vs. 12.9 months; P = 0.64)

There were no significant differences in the median OS between those who received chemotherapy and those did not receive it in the super-elderly group. The OS of patients who received chemotherapy was not significantly prolonged (18.5 vs. 8.4 months; P = 0.33), and a similar trend was observed even in patients with PNI values > 35 (18.5 vs. 12.9 months; P = 0.64). These data are summarized in Fig. 4a, b

## Discussion

The current study showed that low PNI and high CEA levels were poor prognostic factors for mCRC in the super-elderly group and chemotherapy was not a prognostic factor for mCRC in the super-elderly group. Only few studies of super-elderly patients with mCRC, especially with respect to the efficacy of chemotherapy, have been reported. Despite several clinical trials, the efficacy of combination chemotherapy based on oxaliplatin in super-elderly patients remains unelucidated. A previous retrospective study compared the efficacy of FOLFOX in patients aged ≥ 70 years. The study included 3742 patients (614 patients aged ≥ 70 years) and showed no differences according to in age progression free-survival (PFS) (hazard ratio: 0.70, age < 70 years; hazard ratio: 0.65, age ≥ 70 years; P = 0.42) and OS (hazard ratio: 0.77, age < 70 years; hazard ratio: 0.82, age ≥ 70 years; P = 0.79). However, only 15 super-elderly patients were included [24]. Another report evaluated the efficacy and safety of CAPOX-BV (capecitabine plus oxaliplatin and bevacizumab) in patients aged ≥ 75 years. The study included 36 patients (13 patients aged ≥ 80 years), and 21 patients (58.3%) received second-line chemotherapy. The median PFS and OS was 8.8 months (95% CI 6.7–10.3 months) and 20.8 months (95% CI 16.1–26.4 months), respectively [8].

However, there are limited studies of mCRC in the super-elderly group.

The present study showed that the proportion of patients in the super-elderly patients with favorable PS scores (0–1) and good nutritional status were lower than that in the younger group. The super-elderly group was less likely to be treated with chemotherapy than the younger group and had a significantly lower OS than the younger group even in the patients who received chemotherapy. The OS of patients who received chemotherapy tended to be longer than that of those who did not receive chemotherapy; however, there were no significant differences in the super-elderly group. The proportion of super-elderly patients who did not receive treatment was higher than that of younger patients. The proportion of patients treated with chemotherapy was only 28%. In recent reports, the proportion was 22%–48%, and the result did not differ [14, 15, 25]. We identified CEA and PNI in the super-elderly group and resection of primary tumor and chemotherapy and albumin level in the younger group as prognostic factors of mCRC. A previous systematic review showed that CEA was an independent prognostic significance factor [16, 26]. Some previous reports showed that PNI was a prognostic factor of colorectal cancer, resected colorectal cancer, and mCRC [27, 28].

In our study, the median OS of super-elderly patients who received chemotherapy was longer than that of those who did not, although no significant difference was observed (18.5 vs. 8.4 months; P = 0.33). Grade 3 or 4 adverse events in first-line chemotherapy were reported in 66.7%, and the most frequent adverse event ≥ grade 3 reported in the super-elderly group was fatigue (33.3%). Meanwhile, patients who received mono-chemotherapy did not report any grade 3 or higher adverse event during first-line chemotherapy. In contrast, few studies showed that treatment with chemotherapy was significantly effective in prolonging OS in super-elderly patients. In a previous study of chemotherapy under the age of 75, the most frequent adverse event ≥ grade 3 was neutropenia, with grade 3 neutropenia reported in 11.1% of patients; meanwhile, grade 3 fatigue was only reported in 2.8% of patients [8]. In our study, most patients in the super-elderly group (n = 10, 83%) received combination chemotherapy, the most common of which was an oxaliplatin-based regimen. In addition, only 5 (42%) patients and only 1 (8%) patient in the super-elderly group received second-line and third-line chemotherapy, respectively. Studies conducted in Australia showed that the survival of super-elderly patients who received chemotherapy was similar to that of younger patients. In addition, 74.2% of super-elderly patients received single-agent chemotherapy, and 35% and 33% of these patients received second-line and third-line chemotherapy, respectively. The OS of super-elderly patients who received chemotherapy was 19 months [15]. In our study, the efficacy of chemotherapy could not be shown due to the high number of patients with adverse events ≥ grade 3 and low number of patients with third-line chemotherapy.

The current study had some limitations. First, this was a retrospective and single-center study. Therefore, we could not avoid information bias, particularly regarding adverse effects and discuss complications, which are important factors in studies involving elderly patients. Second, it was not possible to discussion about patients who could be treated with effective chemotherapy because the indications for chemotherapy were not clearly defined. Third, the study included only a small number of patients who received chemotherapy. All the super-elderly patients who received chemotherapy had PNI > 35, and this may have made it difficult to determine the efficacy of chemotherapy. Therefore, a prospective study should be performed to determine the efficacy of chemotherapy in super-elderly patients with high PNI values.

## Conclusions

Super-elderly patients with mCRC had worse nutrition status and poorer PS score than younger patients with mCRC, and they had a significantly lower OS than younger patients even though they were treated with chemotherapy. In addition, high CEA levels and low PNI were poor prognostic factors of mCRC in super-elderly patients.

## Supplementary Information

**Additional file 1:** Figure S1: Flowchart of the inclusion and exclusion of patients in the study

**Additional file 2:** Table S1: Summary of the chemotherapy regimens. Chemotherapy regimens received by patients in the first-, second-, and third-line treatments separated according to those aged < 80 and ≥ 80 years.

## Data Availability

The data used and analyzed during the current study available from the corresponding author on reasonable request.

## Abbreviations

CRC: Colorectal cancer
mCRC: Metastatic colorectal cancer
PS: Performance status
CEA: Carcinoembryonic antigen
PNI: Prognostic nutritional index
Cis: Confidence intervals
OS: Overall survival
