# Supplementary figures and images for: Clinical and therapeutic features and prognostic factors of metastatic colorectal cancer over age 80: a retrospective study
Source: BMC Gastroenterol. 2021 May 1;21:199. doi: 10.1186/s12876-021-01791-9 (PMC8088714; doi:10.1186/s12876-021-01791-9)

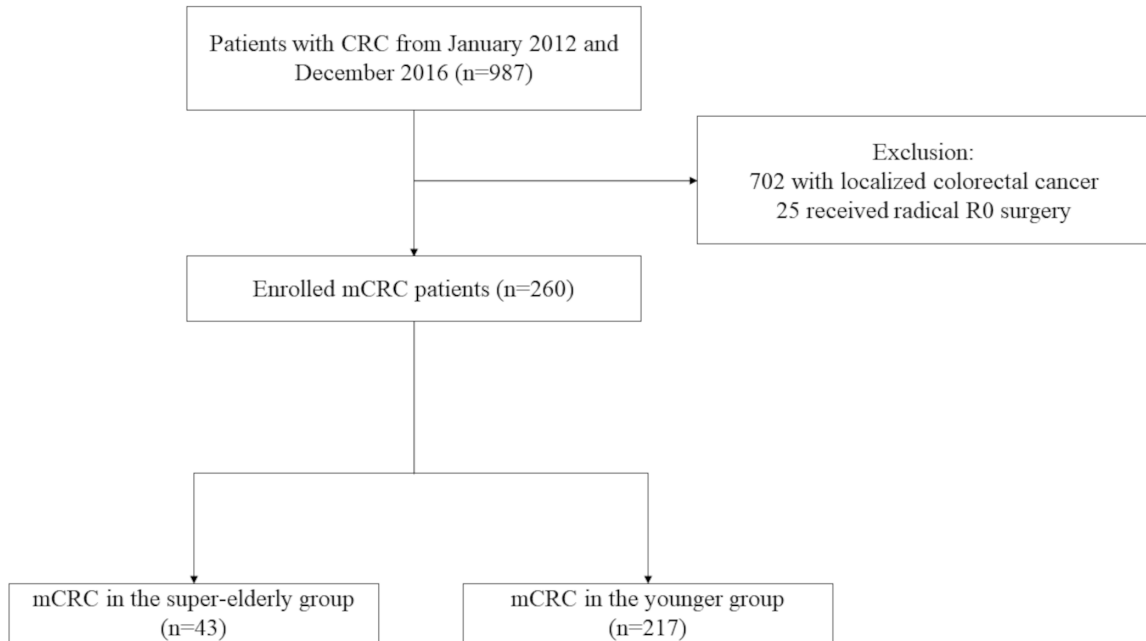

Supplement: Supplementary file 1 — Additional file 1: Figure S1: Flowchart of the inclusion and exclusion of patients in the study [file 12876_2021_1791_MOESM1_ESM.pdf]
